# Effective Treatment of Metastatic Melanoma by Combining MAPK and PI3K Signaling Pathway Inhibitors

**DOI:** 10.3390/ijms20174235

**Published:** 2019-08-29

**Authors:** Synnøve Nymark Aasen, Himalaya Parajuli, Tuyen Hoang, Zichao Feng, Krister Stokke, Jiwei Wang, Kislay Roy, Rolf Bjerkvig, Stian Knappskog, Frits Thorsen

**Affiliations:** 1 Kristian Gerhard Jebsen Brain Tumour Research Centre, Department of Biomedicine, University of Bergen, Jonas Lies vei 91, 5009 Bergen, Norway; 2 Department of Oncology and Medical Physics, Haukeland University Hospital, Jonas Lies vei 65, 5021 Bergen, Norway; 3 Brain Science Research Institute, Shandong University, 44 Wenhuaxi Road, Jinan 250100, China; 4 NorLux Neuro-Oncology Laboratory, Department of Oncology, Luxembourg Institute of Health, 84 Val Fleuri, 1526 Luxembourg, Luxembourg; 5 Section of Oncology, Department of Clinical Science, University of Bergen, 5020 Bergen, Norway; 6 The Molecular Imaging Center, Department of Biomedicine, University of Bergen, Jonas Lies vei 91, 5009 Bergen, Norway

**Keywords:** melanoma, brain metastasis, BRAF, MAPK, PI3K, combined treatment, apoptosis

## Abstract

Malignant melanoma is the most aggressive type of skin cancer and is closely associated with the development of brain metastases. Despite aggressive treatment, the prognosis has traditionally been poor, necessitating improved therapies. In melanoma, the mitogen activated protein kinase and the phosphoinositide 3-kinase signaling pathways are commonly altered, and therapeutically inhibiting one of the pathways often upregulates the other, leading to resistance. Thus, combined treatment targeting both pathways is a promising strategy to overcome this. Here, we studied the in vitro and in vivo effects of the PI3K inhibitor buparlisib and the MEK1/2 inhibitor trametinib, used either as targeted monotherapies or in combination, on patient-derived melanoma brain metastasis cell lines. Scratch wound and trans-well assays were carried out to assess the migratory capacity of the cells upon drug treatment, whereas flow cytometry, apoptosis array and Western blots were used to study apoptosis. Finally, an in vivo treatment experiment was carried out on NOD/SCID mice. We show that combined therapy was more effective than monotherapy. Combined treatment also more effectively increased apoptosis, and inhibited tumor growth in vivo. This suggests a clinical potential of combined treatment to overcome ceased treatment activity which is often seen after monotherapies, and strongly encourages the evaluation of the treatment strategy on melanoma patients with brain metastases.

## 1. Introduction

Melanoma is the most lethal and aggressive type of skin cancer, but only represents around 5% of all cases of skin cancer [1]. Together with non-small cell lung cancer (non-small-cell lung cancer; NSCLC) and breast cancer, melanoma has the highest propensity of all cancers to induce brain metastases. Around 50% of melanoma patients with metastatic disease exhibit cancer spread to the brain, a number that rises to more than 70% at autopsy [2]. The incidence is increasing [3], and despite the use of standard as well as novel therapies, metastatic melanoma is associated with divergent prognoses [4]. Thus, there is still an urgent need for developing new and improved treatment strategies for melanoma patients.

Comprehensive surveys of the genetic landscape of malignancies have shown that melanomas have the highest mutation frequency of all cancers analyzed [5]. In particular, the mitogen activated protein kinase (MAPK) and the phosphoinositide 3-kinase (PI3K) signaling pathways are commonly altered in melanoma [6]. Up to 90% of melanomas display an aberrant activation of the MAPK pathway, leading to cell cycle deregulation and inhibition of apoptosis [7]. The most common somatic mutations are activating point mutations in the v-Raf murine sarcoma viral oncogene homolog (BRAF; 35–50% of melanomas) and neuroblastoma RAS viral oncogene homolog (NRAS; 10–25%), as well as loss-of-function mutations affecting neurofibromin 1 (NF1; ~15%) [8]. The substitution of valine for glutamic acid (V600E) is found in approximately 90% of all BRAF mutations (BRAF^V600E^) [9]. Activated BRAF leads to the down-stream activation of protein mitogen-activated protein kinase kinase (MEK) by inducing phosphorylation of the serine residues. Activated MEK triggers the serine/threonine kinase extracellular signal-regulated kinase (ERK) by phosphorylation of the Thr-Glu-Tyr motif. The phosphorylated ERK translocates into the nucleus and regulates gene expression of over 50 substrates leading to progression of cancer [10].

The PI3K signaling pathway is also frequently activated in melanoma, often as a consequence of mutations or loss of phosphatase and tensin homolog (PTEN); an inhibitor of protein kinase B (Akt), and further dysregulation of Akt expression [11]. This pathway plays a key role in inducing drug resistance after treatment of melanoma patients with BRAF/MEK inhibitors, and is thus a decisive target for melanoma therapy [12]. More specifically, PI3K activation has been highlighted as a particularly important driver of metastases in the brain [13]. Furthermore, it has been shown that PI3K signaling may result in therapeutic resistance in BRAF^V600E^ mutated metastatic melanoma by modulating MAPK signaling and altering MEK activity [14]. Inhibition of one of the pathways often upregulates the other, thereby inducing resistance to therapy [15].

Mutations in MEK can induce resistance to BRAF inhibition; however, combining a MEK inhibitor with a PI3K inhibitor has been found to overcome this resistance [16]. Thus, a combined PI3K and MEK inhibition can ensure suppression of downstream proliferation, inhibition of growth, and survival signals in tumors. The combination of two such inhibitors can, therefore, enhance toxicity. Trametinib is one of the most effective allosteric inhibitors of MEK1/MEK2 activation and kinase activity [17]. The U.S. Food and Drug Administration (FDA) has approved trametinib (GSK1120212) for treatment of BRAF-mutant metastatic melanoma [18], and a phase III clinical trial using trametinib in melanomas with BRAF^V600E^ or BRAF^V600K^ mutations showed promising responses [19]. Furthermore, more than 30 small molecule PI3K inhibitors have entered clinical trials [20]. A common disadvantage is that they may not effectively downregulate all isoforms of PI3K [21]. Buparlisib (BKM120) is an oral pan-class I PI3K inhibitor targeting all isoforms of PI3K [22]. The anti-proliferative, pro-apoptotic, and antitumor activity of buparlisib has been demonstrated preclinically [23] and in clinical trials [24].

In the present study, we investigated the combinatorial effect of the targeted therapies buparlisib and trametinib in vitro and in vivo on patient-derived melanoma cell lines of brain metastatic origin. Through mechanistic investigations, we show that combined treatment using trametinib and buparlisib was more effective than monotherapy regarding increasing tumor cell death, which could indicate a way of overcoming ceased treatment effect, as is often observed in the clinic. 

## 2. Results

### 2.1. Combined Trametinib and Buparlisib Treatment Is More Effective Than Monotherapies In Vitro

**Figure 1:**
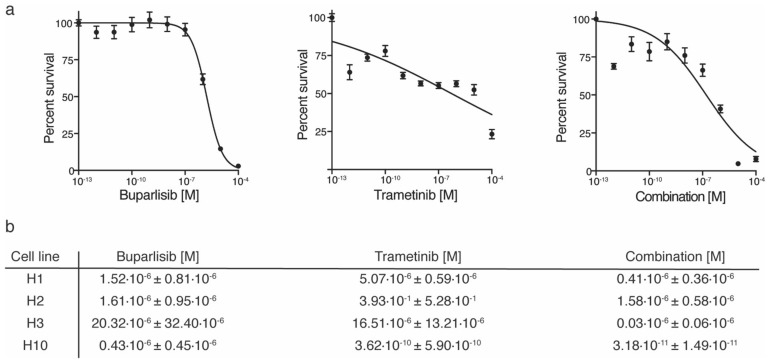
Cell survival curves of cells grown as monolayers after treatment with buparlisib, trametinib or a combination with increasing drug concentrations (0.000001 μM–100 μM). (**a**) representative graphs of H1 cells treated with increasing drug concentrations for 72 h, with either buparlisib, trametinib, or combined treatment; (**b**) table showing the IC_50_ doses, the doses at which 50% of the cells were growth inhibited, for all four cell lines studied. The experiments were performed in triplicate (*n* = 6 per experiment per drug concentration).

Assessing the effects of trametinib and buparlisib monotherapies as well as the combination of the two, we used four different patient-derived melanoma cell lines; H1, H2, H3 and H10. All four were of brain metastatic origin and all four harbored BRAF mutations (H3 harbored BRAF^L577F^ while H1, H2 and H10 harbored the canonical BRAF^V600E^). Treating the cells with buparlisib reduced cell viability to a greater extent than trametinib in both H1 and H2 cells (Figure 1a,b). Combined treatment was more effective than either monotherapy in H1 cells, whereas combined treatment did not further increase cell death in H2 cells (Figure 1b, Supplementary Figure S1). The H10 cells were more sensitive to trametinib than buparlisib, and combined treatment was more effective than either of the monotherapies (Figure 1b, Supplementary Figure S1). The BRAF^L577F^ mutated H3 cells were more resistant to single drug treatment than the other BRAF^V600E^ mutated cell lines. Nevertheless, combined treatment was more effective than the single drug treatments (Figure 1b, Supplementary Figure S1). These results were used as a baseline for further in vitro experiments. 

### 2.2. Treatment with Buparlisib and Trametinib Decreases Target Protein Expressions

**Figure 2:**
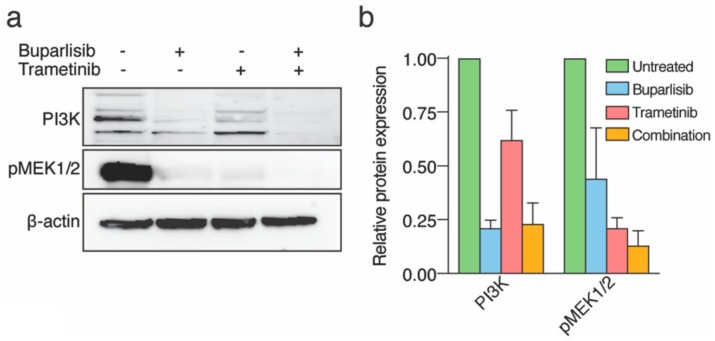
Protein expression of cell lysates after in vitro treatment with 10 μM buparlisib, 10 μM trametinib or combination (5 μM + 5 μM). (**a**) Western blots of lysates from H1 cells showing the expression of PI3K and MEK1/2; (**b**) quantification of PI3K and MEK1/2 expression relative to β-actin.

To validate the cellular expression of the two signaling pathways upon therapeutic inhibition, lysates from H1, H2, H3 and H10 were prepared for Western blot analysis. Untreated H1, H2, H3 and H10 cells all expressed PI3K activation and MEK1/2 phosphorylation (Figure 2a,b and Supplementary Figure S2). The expression of PI3K activity and MEK1/2 phosphorylation decreased after single monotherapies, however, combined treatment most effectively downregulated the protein expressions.

### 2.3. Combined Treatment Inhibits 2D and 3D Colony Formation More Effectively Than Single Drug Treatment

**Figure 3:**
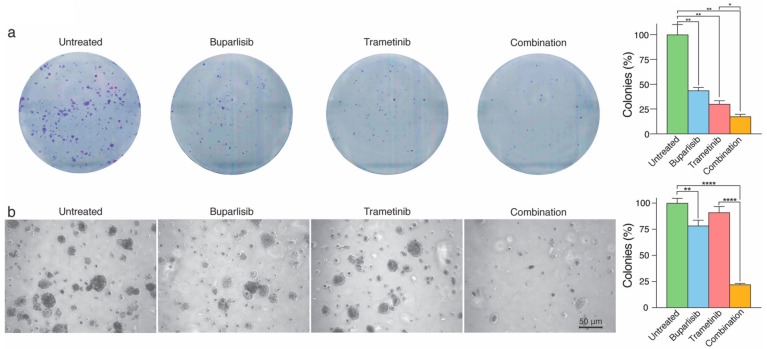
In vitro colony formation of H1 cells after pre-treatment with buparlisib and trametinib. (**a**) representative images of H1 cells pre-treated with 10 μM buparlisib, 10 μM trametinib or a combination (5 μM buparlisib + 5 μM trametinib) grown as colonies. The colony formation was scored and quantified as seen in the graph to the right; (**b**) representative images of H1 cells pre-treated with corresponding drug concentrations seeded into low melting point agarose and incubated for 21 days. Scale bar = 50 μM. The percentage area covered by the spheroids within the total visual field was quantified as seen to the right. The experiments were performed in triplicate (*n* = 4 images). Abbreviations: *: *p* < 0.05, **: *p* < 0.01 and ****: *p* < 0.0001.

To study whether the therapeutic strategy inhibited cell growth after pre-treatments as an indication of colony formation, we carried out clonogenic assays in 2D and 3D. Out of the four cell lines, only H1 and H2 cells grew as colonies in 2D. Cells pre-treated with buparlisib developed 43.7% colonies compared to untreated cells, and H1 cells pre-treated with trametinib developed 30% colonies (for both, *p* < 0.01; Figure 3a). Combination treatment was most effective, as only 17.5% colonies developed, compared to untreated cells (*p* < 0.05 compared to trametinib treatment; Figure 3a). For H2 cells, single drug treatment with buparlisib was more effective than trametinib, whereas combinatorial treatment again was more efficient than single drug treatments (*p* < 0.0001 compared to untreated cells, Supplementary Figure S3).

Only H1 cells grew as colonies in a 3D anchorage-independent culture environment. H1 cells pre-treated with trametinib and grown in the same conditions covered in comparison around 91.0% of the field of view. Cells treated with buparlisib covered around 78.4% of the total area (*p* < 0.01, compared to untreated cells), while the area covered after combined treatment was around 22.1% (*p* < 0.0001, compared to untreated cells; Figure 3).

### 2.4. Tumor Cell Migration and Directional Cell Migration towards a Chemo-Attractant Is Hampered by Combination Treatment

Since we observed reduced clonogenic growth after pre-treatment in monolayer and anchorage-independent cell cultures, we studied the migratory capacity of the metastatic cells after treatment. Accordingly, we carried out two different migration assays: a scratch wound assay and a trans-well assay.

**Figure 4:**
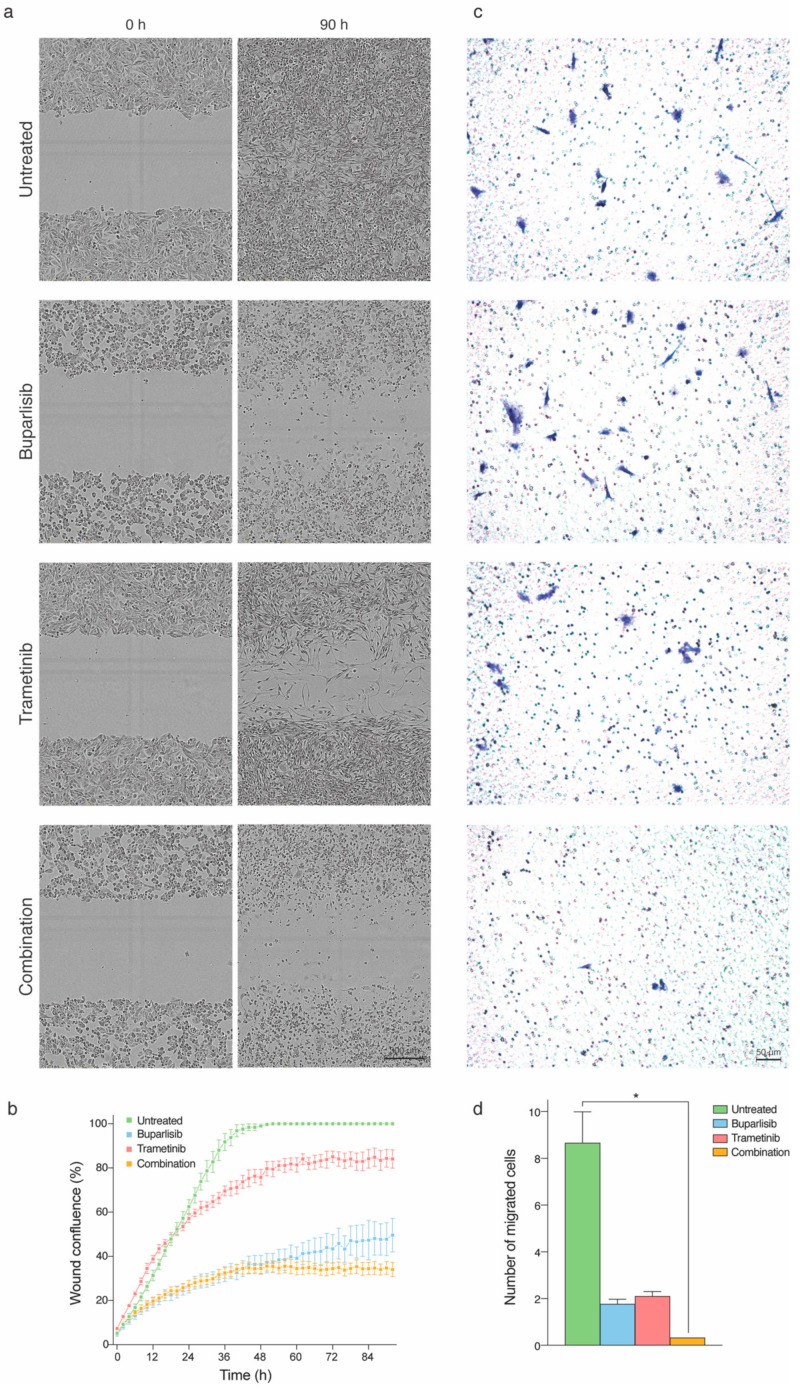
Migration of H1 cells during and after pre-treatment with 10 μM buparlisib, 10 μM trametinib or a combination (5 μM buparlisib + 5 μM trametinib). (**a**) representative micrographs of H1 cells before (0 h) and at completion (90 h) of the scratch-wound experiment. The drugs were added at initiation of the time-lapse experiment. Scalebar = 300 μm; (**b**) quantification of wound confluence throughout the time-lapse across the different treatment groups (*n* = 6 per drug concentration); (**c**) representative images of migrated H1 cells pre-treated with buparlisib, trametinib or a combination. Scalebar = 50 μm; (**d**) quantification of H1 cells allowed to migrate towards a chemo-attractant for 48 h. The experiment was carried out in triplicate (*n* = 3 representative fields of view per experiment). Abbreviations: *: *p* < 0.05.

During the scratch wound assay, the cells were under constant exposure to the respective drugs. The wound confluence measured during the experiments was scaled to percentage during analysis. Across all cell lines, the most efficient treatment was a combination of buparlisib and trametinib, followed by buparlisib and then trametinib (Figure 4, Supplementary Figure S4). After approximately 50 h, the wound was completely closed for untreated H1 cells (Figure 4a,b). After 90 h, none of the other treatment groups had managed to regrow the wound completely. The lowest percentage of confluence was observed after combined treatment of the H1 cells (Figure 4a,b). Among the other cell lines used, H3 was the only one that was completely regrown into the wound upon completion of the experiment at 90 h (Supplementary Figure S4b). H10 cells were the most sensitive to all treatments (Supplementary Figure S4c).

In the trans-well assay, the evaluation of cell migration was carried out after pre-treating the cells with the respective drugs for 24 h. In contrast to the scratch-wound assay, the trans-well assay revealed that trametinib was a more effective monotherapy than buparlisib regarding inhibiting migratory properties of cells across all cell lines. In addition, here, the combination treatment was more effective than either of the monotherapies (Figure 4c,d and Supplementary Figure S5).

### 2.5. Melanoma Cell Morphology Is Altered upon Combination Treatment

The morphology of the brain metastatic melanoma cells was observed to change upon treatment with buparlisib and trametinib. For all four cell lines, an elongated morphology was observed after treatment with trametinib, whereas buparlisib or combination treatment resulted in a circular cell shape with reduced attachments (Supplementary Figure S6). 

### 2.6. Combined Treatment Induces More Apoptosis Than Single Drug Treatment

**Figure 5:**
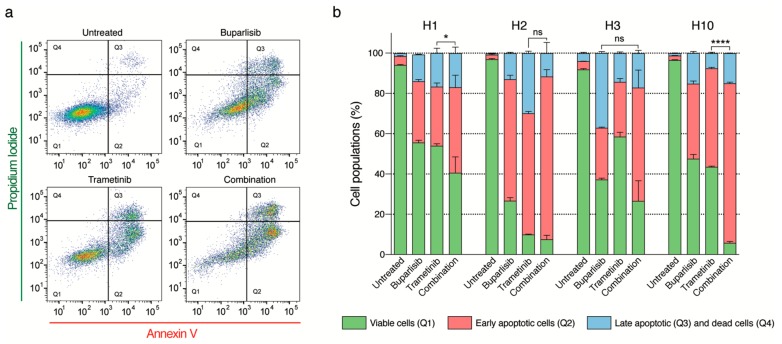
Flow cytometric analysis of apoptosis after treatment of melanoma brain metastasis cells with buparlisib and trametinib. (**a**) Representative dot plots of H1 cells treated with 10 μM buparlisib, 10 μM trametinib or combination (5 μM buparlisib and 5 μM trametinib). Annexin V labels apoptotic cells, while Propidium Iodide labels necrotic cells. (**b**) Quantification of the percentage of viable, apoptotic and necrotic cells for the H1, H2, H3 and H10 cell lines. The experiments were done in triplicate (n = 3 per experiment per drug concentration). Abbreviations: Q1: viable cells, Q2: cells in early apoptosis, Q3: late apoptotic cells, Q4: necrotic cells, ns: not significant, *: *p* < 0.05 and ****: *p* < 0.0001.

To assess whether the altered morphology could indicate apoptosis, we studied this by flow cytometry using a PI and annexin V assay after treating the cells for 72 h (Figure 5). Combination treatment reduced cell viability across all four cell lines, by entering early or late apoptosis (Figure 5). For untreated H1 cells, around 94% of the cells were viable (Figure 5a,b). Single drug treatment decreased the number of viable cells to around 55%, while combined treatment decreased the number of viable cells to around 40%. Approximately 10% of the H2 cells survived after trametinib treatment, and almost 30% of the cells were healthy after being exposed to buparlisib. Combined treatment resulted in a 7% cell survival (Figure 5b). For the H3 cell line, cell survival after exposure to single drug treatment was around 58% (trametinib) and 37% (buparlisib). In addition, here, combined treatment effectively reduced cell survival, compared to monotherapies to around 25% (Figure 5b). Between 40–50% of H10 cells treated with single drugs survived the treatment. Here, combined drug treatment effectively reduced the number of healthy cells to approximately 6% (Figure 5b). For H1, H2 and H10, trametinib was the most effective monotherapy. For H3 cells, buparlisib was the most effective monotherapy.

**Figure 6:**
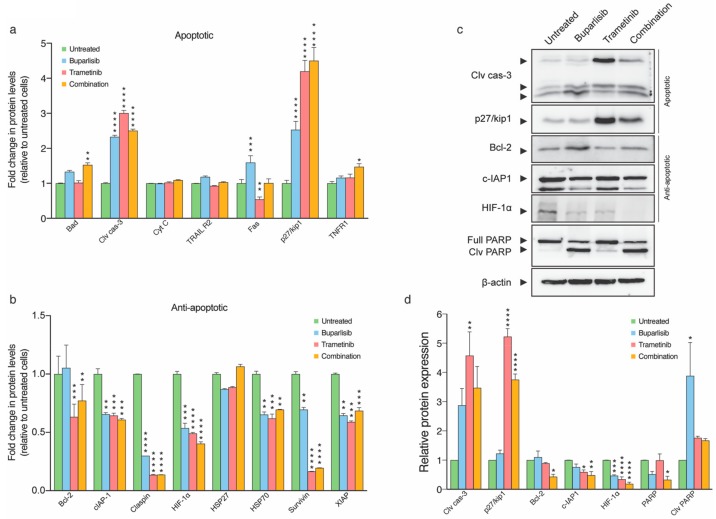
Expression of apoptosis associated proteins after treatment of H1 cells with 10 μM buparlisib, 25 nM trametinib or a combination (5 μM buparlisib + 12.5 nM trametinib). (**a**) graphic presentation of results from the apoptosis array, showing expression of key molecules involved in apoptosis in H1 cells treated with 20 μM buparlisib, 50 nM trametinib, or 10 μM buparlisib + 25 nM trametinib, for 72 h; (**b**) graphic representation of key molecules involved in anti-apoptotic activity in H1 cells after buparlisib and trametinib treatment; (**c**) Western blots showing the expression of selected apoptotic and anti-apoptotic proteins in addition to PARP; (**d**) quantification of Western blot results relative to β-actin. Abbreviations: Clv cas-3: cleaved caspase-3, cyt C: cytochrome C, full: full length PARP, clv: cleaved, *: *p* < 0.05, **: *p* < 0.01, ***: *p* < 0.001 and ****: *p* < 0.0001.

To gain a more detailed, mechanistic understanding of the observed apoptosis, we conducted an apoptosis array experiment (Figure 6a, b). Both monotherapy and combination treatment resulted in statistically significant increases in expressions of the apoptotic proteins Bad, cleaved caspase-3, p27/kip1 and tumor necrosis factor receptor 1 (TNFR1). There were also tendencies of increased expression of cytochrome-C, TRAIL R2 and Fas in the monotherapies (Figure 6a). Expression of key inhibitors of apoptosis such as Bcl-2, cIAP-1, claspin, HIF-1α, HSP70, survivin and XIAP were significantly downregulated by both the monotherapies and the combination treatment. Tendencies of decreased expression after monotherapies were also seen in HSP27 (Figure 6b).

A selection of key apoptotic and anti-apoptotic proteins identified from the apoptosis array (Figure 6a,b) was then verified by quantitative Western blot analyses (Figure 6c,d). Statistically significant increases in protein levels of cleaved caspase-3 were found for lysates from H1 cells treated with trametinib (*p* < 0.01). Increased expression of p27/kip1 was seen for cells treated with trametinib and combination therapy (both *p* < 0.0001). Statistically significant decreases were also detected for Bcl-2 in the combination group (*p* < 0.05) and for HIF-1α across buparlisib, trametinib and combination group (*p* < 0.001, 0.0001 and 0.0001, respectively). PARP, which is considered a hallmark of apoptosis, was also downregulated in the combination group (*p* < 0.05; Figure 6c,d).

### 2.7. The Combinatorial Approach Is More Efficacious Than Single Treatments In Vivo

**Figure 7:**
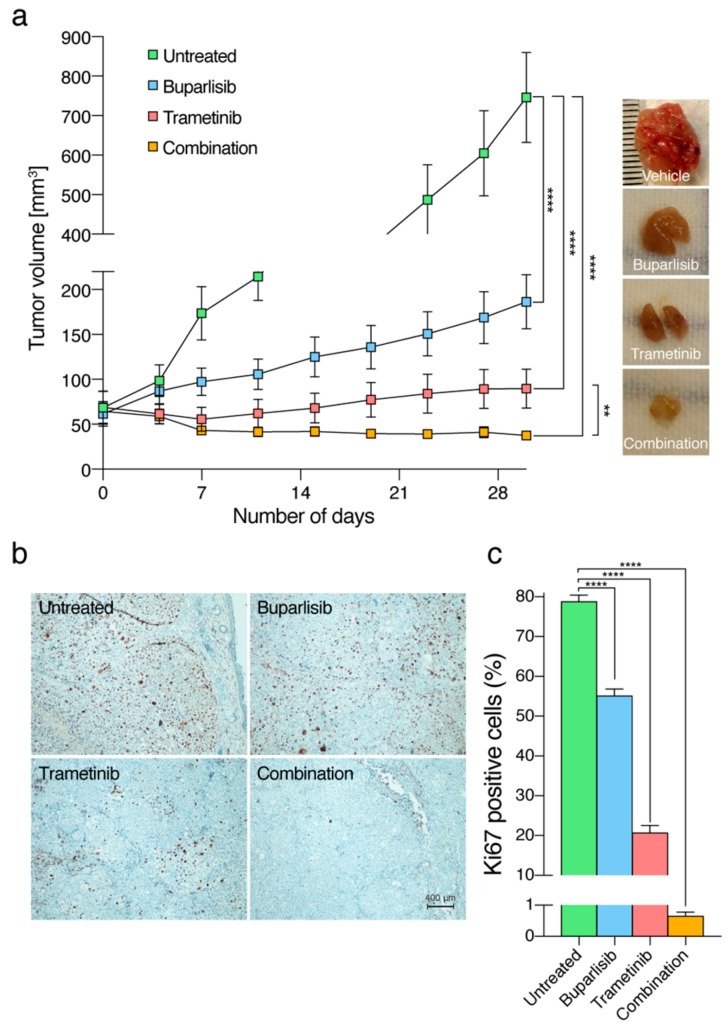
Inhibition of in vivo tumor growth after treatment with 50 mg/kg buparlisib and 1 mg/kg trametinib. (**a**) tumor volumes plotted as a function of time to assess tumor growth in response to therapy. The tumors were measured once a week and at the end-point of the experiment (30 days). Representative images of subcutaneous tumors after termination of the experiment can be seen on the right (*n* = 8 mice in each treatment group); (**b**) immunohistochemistry sections from representative mice from each treatment group stained with antibodies against Ki67 (imaged with a 10× objective); (**c**) quantification of the number of Ki67 positive cells in each treatment group. Abbreviations: **: *p* < 0.01 and ****: *p* < 0.0001.

The finding that combined treatment with buparlisib and trametinib in vitro was more efficient than single treatments was validated in vivo in NOD/SCID mice with subcutaneous H1_DL2 derived tumors. At the endpoint of the experiment (after 30 days), the average tumor volume in the combination group was 37.7 mm^3^, in the trametinib group 89.7 mm^3^, in the buparlisib 186.3 mm^3^ and the tumors in the vehicle group 746.7 mm^3^ (Figure 7a). Compared to vehicle treated tumors, buparlisib, trametinib and combination treated mice showed a statistically significant reduction in total tumor volumes (all *p* < 0.0001). When the combination treatment group was compared to the trametinib group, there was also a statistically significant difference (*p* < 0.01; Figure 7a). 

The number of Ki67 positive cells was quantified from histological sections from tumor tissue across all treatment groups. There were statistically significant differences between vehicle treated tumors and all treatment groups (all *p* < 0.0001; Figure 7b,c).

## 3. Discussion

In view of previous research showing a synergic effect of combining MEK1/2 and PI3K inhibitors [25], we evaluated the therapeutic activity of the combinatorial approach in BRAF^V600E^ and BRAF^L577F^ mutated melanoma cells of brain metastatic origin. Although combined treatment with buparlisib and trametinib have been carried out previously for a variety of cancers [26,27,28,29], the combined use of these two drugs for treatment of brain metastatic melanoma has not been investigated to date. Thus, our results should encourage further studies to develop this to be a novel treatment strategy for patients with melanoma brain metastasis.

MAPK inhibition has previously been associated with acquired treatment resistance [18]. The PI3K pathway represents a second essential signaling pathway in melanoma progression, as brain metastasis are often PI3K activated [12,13]. It has previously been shown that activation of the PI3K pathway can mediate resistance to MEK inhibitors [30]. In addition, inhibition of the PI3K pathway can sensitize melanoma cells towards chemotherapies [31]. PI3K inhibition using buparlisib has previously been demonstrated as a promising treatment strategy in BRAF mutated brain metastases in nude mice [32]. In a phase Ib clinical trial treating cancer patients (nine of the patients had cutaneous melanoma) with buparlisib and trametinib, the treatment was shown to be well tolerated in the short term [29].

In our experiments, trametinib downregulated the expression of MEK1/2 in all BRAF^V600E^ cell lines. In the BRAF^L577F^ mutated cell line H3, there was no reduction in protein expression, which may indicate resistance against trametinib. Upon treatment with buparlisib, however, PI3K was downregulated in all cell lines, and the combinatorial approach reduced the protein expression in all cell lines, regardless of BRAF status. Our results suggest that cell lines not harboring the BRAF^V600E^ mutation may be more susceptible to PI3K inhibition. This is in line with previous research, where a decrease in Akt phosphorylation and hence buparlisib activity in both BRAF^WT^ and BRAF^V600E^ mutated melanoma cells was demonstrated [32]. Our results also indicate a higher degree of resistance towards MEK inhibition and that adding PI3K inhibitors has the potential to abrogate trametinib resistance. We emphasize that the mutation found in the H3 cells has not been reported in the literature to date, so the oncogenic potential of this mutation is not clear.

Of the cell lines used in this study, only H1 and H2 cells were able to grow as monolayer colonies when pre-treated and thereafter seeded in 6-well plates. For both cell lines, there was a statistically significant difference in growth between untreated and combination treated cells (both *p* < 0.0001). For the H1 cells, the most efficient drug was trametinib, whereas buparlisib inhibited cell growth more in H2 cells. When grown in an anchorage-independent environment, the effects of the monotherapies were greatly reduced, as compared to the monolayer experiments. This is likely explained by the more realistic representation of the in vivo situation in 3D [33].

It has formerly been reported that buparlisib can alter the cell morphology by means of reduced focal adhesion and changes in the microtubule dynamics [27]. In our study, all cell lines exhibited traits that could be consistent with the onset of apoptosis after buparlisib treatment, which was also seen after combined treatment. We also observed a different morphology in cells treated with trametinib; however, this change resembled an epithelial-mesenchymal transition (EMT), which is associated with loss of apicobasal polarity and stable cell-cell adhesion. This has also been reported previously after treatment with MAPK inhibitors of melanoma cells [28] and NSCLC cells [26]. Our flow cytometry experiments showed that, for all BRAF^V600E^ mutated cell lines (H1, H2 and H10), trametinib induced more apoptosis than buparlisib, and combination treatment further enhanced the effect. In addition, here, H3 was more resistant to trametinib and more susceptive to PI3K inhibition. 

As mentioned initially, melanoma has a strong tendency to metastasize to the brain. It is acknowledged that metastasis is a dynamic process dependent on the ability of tumor cells to migrate in the microenvironment [34]. Scratch wound assays evaluate the ability of cells to migrate and close an induced wound when grown as a monolayer, whereas trans-well assays give information on the propensity of cells to detect and subsequently migrate towards a chemo-attractant through a physical barrier [35]. During constant exposure to the drugs in the scratch wound assay, buparlisib inhibited cell migration more than trametinib. Interestingly, it has previously been shown that a MEK inhibitor was able to promote cell migration, despite drug-induced cell death in a dose-dependent manner [36]. Concerning the trans-well assay, measuring cell migration towards a chemoattractant, pre-treating cells with trametinib seemed to inhibit cell migration more than buparlisib. This may indicate that trametinib exhibits a longer lasting anti-tumor effect compared to buparlisib in vitro. It could also suggest that melanoma cells are less likely drawn towards a chemoattractant after inhibition of the MAPK compared to the PI3K signaling pathway. In both experimental layouts, combined treatment was the most effective across all cell lines. 

Combined treatment upregulated the expression of the four apoptotic markers bad, cleaved caspase 3, p27/kip1 and TNFR1. The results for cleaved caspase 3 and p27/kip1 were verified by Western blots. Bad is a member of the Bcl-2 family, a substrate of Akt and represents a converging link between inhibition and promotion of apoptosis [37]. Caspase 3 is known as the master regulator of apoptosis and has interestingly been shown to regulate the repopulation of tumors after apoptosis has occurred following treatment [38]. Across all groups, we found a substantial increase in the levels of the cyclin-dependent kinase inhibitor p27/kip1, whose inhibition is characteristic for malignant growth and PI3K inhibition is linked to p27/kip1 inactivation [39]. The tumor necrosis factor receptor (TNFR) is activated by cytokines belonging to the TNF protein family. Upon receptor mediated activation, apoptosis might be induced. Therapeutic MAPK inhibition is associated with acquired drug resistance [40], which may be mediated through increased PI3K signaling [12]. The PI3K signaling pathway regulates the expression of anti-apoptotic proteins such as Bcl-2 [41], cIAP-1 [42], claspin [43], HIF-1α [44], Hsp27 [45], Hsp70 [46], XIAP [47] and surviving [48]. In general, we also found reduced expression levels in the anti-apoptotic proteins after treatment. Expression levels of Bcl-2, cIAP-1 and HIF-1α were verified by Western blots. For all three markers, combinatorial treatment resulted in statistically significant reductions in protein levels. Reduced levels of Bcl-2 subsequent trametinib and combination treatment is in line with previous research [49]. Furthermore, high tumor levels of HIF-1α have been associated with apoptosis and higher survival rates [50]. HIF-1α is also known to mediate the expression of vascular endothelial growth factor (VEGF) and promote aggressive tumor growth [44]. cIAP-1, XIAP and survivin are all members of the inhibitor of apoptosis (IAP) protein family [51]. Survivin expression is linked to several aberrant activations associated with cancer growth, such as, for instance, the PI3K and MAPK signaling pathways [48]. Finally, PARP cleavage is also a well-known hallmark of apoptosis [52]. Our results by Western blot indicated cleaved PARP in all groups; however, the difference was only statistically significant in buparlisib treated cells. 

In the present work, subcutaneous melanoma tumors derived from brain metastases in NOD/SCID mice were effectively treated with buparlisib combined with trametinib. In general, MEK1/2 inhibitors are well tolerated in preclinical doses up to 1030– mg/kg [53]. Trametinib doses around 1 mg/kg have been frequently used in vivo [54]. In our in vivo experiments, we used 1 mg/kg, which significantly reduced the tumor burden in the monotherapy group receiving trametinib. Furthermore, buparlisib has been administered daily from 2 to 50 mg/kg preclinically [27,55]. Based on previous reports, we chose to use 50 mg/kg in the single drug group, which was tolerated well in vivo. Drawn from our animal experiments, monotherapy with trametinib seemed to be more efficient than buparlisib on H1_DL2 tumors. This is in contrast to some of the in vitro experiments with H1 cells; however, some of these exhibited a constant drug exposure to the tumor cells, while, in the preclinical study, the drugs were administered once per day, enabling time for drug clearance between treatments [56]. Thus, the results suggest that a constant exposure to trametinib is needed for optimal effect of the drug. 

Despite using a subcutaneous tumor model, our results can serve as a proof-of-principle for brain metastatic melanoma as well. In case of a brain metastasis model, buparlisib is expected to cross an intact blood-brain barrier (BBB) [27]. However, a technique for delivering trametinib across the BBB would have to be employed, as the relatively large molecular weight (615.39 Da) and the presence of efflux pumps at the site of the BBB discourages its ability to enter the brain [57]. Thus, we used a subcutaneous model as proof-of-principle to ensure combined drug uptake upon oral administration. It should, however, be noted that, in a clinical setting, the BBB is normally disrupted in brain metastases larger than 2–3 mm [58], which should facilitate penetrance of trametinib into the brain metastatic lesions. 

## 4. Materials and Methods 

### 4.1. Cell Lines and Cell Culture

Written consent was obtained from the patients before tumor material was collected and subsequently used to prepare cell lines. The Regional Ethical Committee (REC Number 013.09, approved 21.04.2013) and the Norwegian Directorate of Health (NSD Number 9634, approved 25.03.2009) approved the tissue collection and biobank storage of tumor biopsies and derived cell lines. Cell line authentication was verified by short tandem repeat (STR) fingerprinting.

The H1, H2, H3 and H10 cell lines were established in our laboratory from patient biopsies of human melanoma brain metastases. The BRAF mutation status of the H1, H2, H3 and H10 cell lines was investigated by performing massive parallel sequencing of the tumor DNA, according to published protocols [59]. The H1, H2 and H10 cell lines are V600E mutated, while the H3 cells are L577F mutated. 

The H1 cells were transduced with two lentiviral vectors, encoding Dendra (a green fluorescent protein (GFP) variant) and luciferase to obtain the H1_DL2 cell line. Flow cytometric isolation of cells by GFP expression was performed (BD FACS Aria, Becton Dickinson, Franklin Lakes, NJ, USA).

All cells were grown in Dulbecco’s modified eagles medium (DMEM; Sigma-Aldrich Inc., St. Louis, MO, USA), supplemented with 10% heat-inactivated new-born calf serum (Thermo Fischer Scientific, Waltham, MA, USA), 5 μg/mL Plasmocin (Invivogen, Toulouse, France), 2% L-glutamine (BioWhittaker, Verviers, Belgium), penicillin (100 IU/mL) and streptomycin (100 μL/mL) (BioWhittaker). The cells were cultured in a standard tissue incubator at 37 °C, 100% humidity and 5% CO_2_, and trypsinated once they attained 75% confluency using 0.25% Trypsin/EDTA (BioWhittaker).

### 4.2. Drugs

Buparlisib (BKM-120) and trametinib (GSK1120212) were purchased from ChemieTek (Indianapolis, IN, USA). Both drugs were dissolved in dimethylsulfoxide (DMSO) and stock concentrations of 100 mM buparlisib and 80 mM trametinib were stored at −20 °C in aliquots until further use.

### 4.3. Animals

Female non-obese diabetic/severe combined immunodeficient (NOD/SCID) mice were purchased from Envigo (Gannat, France). The animals were bred and maintained in our animal facility certified by the Association for Assessment and Accreditation of Laboratory Animal Care International. They were fed a standard pellet diet and provided water ad libitum. The National Animal Research Authority approved all animal procedures (application #11655, approved 07.03.2017). 

Anesthesia was induced with 3% sevoflurane (Abbott Laboratories Ltd., Berkshire, UK) in oxygen and maintained with 1.5% sevoflurane in oxygen during the injection procedures.

### 4.4. Cell Viability Assay

Cell proliferation upon treatment with buparlisib and/or trametinib was studied using a resazurin assay, as previously described [6]. All cell lines were seeded at a density of 5 × 10^3^ cells/well in 200 μL culture medium in 96 well plates (Nunc, Roskilde, Denmark) and treated with buparlisib or trametinib (0, 0.000001, 0.00001, 0.0001, 0.001, 0.01, 0.1, 1, 10 and 100 µM), or a combination of both these treatments for a period of 72 h. After treatment, 20 µl of 0.01 mg/mL resazurin (Sigma-Aldrich Inc., St. Louis, MO, USA) diluted in phosphate buffered saline (PBS) was added to each well and incubated for 4 h at 37 °C. The absorbance was measured at dual mode 560/590 using a scanning multi-well spectrophotometer (Victor 3 1420 multi-label counter, Perkin Elmer, Waltham, MA, USA). Each treatment was performed in triplicate (*n* = 6 per experiment per drug concentration). IC_50_ concentrations were drawn from the results, i.e., the drug concentration at which 50% of the cell growth was inhibited. 

### 4.5. Clonogenic Assays

In order to measure the potential loss of reproductive integrity of cancer cells, clonogenic assays were performed. The cell lines H1, H2, H3 and H10 were seeded at a density of 5 × 10^6^ cells/well in 2 mL culture medium in 6-well plates (Nunc) and allowed to reach 75% confluency. The cells were then treated with 20 µM buparlisib, 50 nM trametinib or combined therapy (10 µM buparlisib and 25 nM trametinib) for 72 h. The drug concentrations were optimized for this experiment. Untreated cells were maintained as controls. After treatment, the cells were washed with PBS and trypsinated using 0.25% Trypsin/EDTA. The cells were centrifuged at 900 rpm for 4 min, the supernatant was discarded and the cells were resuspended in fresh culture medium. The cells were then counted using a Countess^®^ automated cell counter (Invitrogen, Carlsbad, CA, USA) according to the manufacturers protocol. 1000 cells from each treatment were seeded in fresh 6 well culture dishes (Nunc) in 2 mL culture medium and allowed to grow for 12 days. After 12 days, the culture medium was removed from the wells and the cell layer was washed with cold PBS. The cells were thereafter fixed with prechilled methanol at −20 °C for a period of 10 min. The methanol was removed and plates were left to dry. The cells were then stained using 0.5% crystal violet in 25% methanol in water for 10 min. The stain was removed, the cells were washed three times with milliQ water and the plate was allowed to dry. Colonies consisting of more than 50 cells were counted as surviving colonies. The number of untreated colonies were scaled to 100%. The experiment was performed in duplicate (*n* = 3 per experiment per treatment group).

The cells were also grown in an anchorage-independent environment, using a 3D colony formation assay. 7.5 × 10^5^ cells were seeded in T75 culture flasks (Nunc) and incubated until approximately 60% confluency. The culture medium was then replaced with either fresh culture medium (negative control), fresh culture medium containing 10 μM buparlisib, 10 μM trametinib or a combination of 5 μM buparlisib and 5 μM trametinib. After 72 h of incubation, the cells were washed with PBS, trypsinated and centrifuged (900 rpm for 4 min). The cell number was adjusted to a density of 8 × 10^4^ cells/mL and mixed 1:1 with 0.6% low melting point agarose (Sigma-Aldrich). The cells were then seeded on top of 100 μL of cooled 0.6% Difco noble agar (BD Biosciences) in a 96-well plate (Nunc) and kept at 4 °C for 20 min. The low melting point agarose layer with cells was then overlaid with 100 μL of fresh culture medium, and the plate was incubated for 21 days. Colony formation was analyzed as growth area covered by untreated H1 cells after 21 days and scaled to 100% using micrographs taken with a Nikon TE2000 inverted microscope (Nikon Instruments Inc., Melville, NY, USA). The analysis was carried out using GraphPad Prism v7 (GraphPad Software Inc., San Diego, CA, USA). The experiment was performed in duplicate (*n* = 3 per experiment per drug treatment group).

### 4.6. Cell Migration

To study the effects of treatment on cell motility, two cell migration experiments were carried out. In the first experiment, H1, H2, H3 and H10 cells were plated at a density of 5 × 10^5^ cells/well in 2 mL culture medium in 6-well plates (Nunc). After 24 h, the cells were pre-treated with 10 µM buparlisib, 10 µM trametinib or combined therapy (5 µM buparlisib and 5 µM trametinib) for 24 h. An untreated well was maintained as a negative control. After incubation with drugs, the cells were washed twice with PBS, trypsinated and seeded at a density of 1 × 10^3^ cells/well in Thinsert^TM^ cell culture inserts (Greiner Bio-One GmbH, Frickenhausen, Germany) in 24-well plates (Nunc). The lower chambers were filled with 500 µL culture medium containing 30% serum as chemoattractant. After incubation in 48 h, the inserts were removed from the wells and washed with PBS to remove unbound cells. The inserts were fixed with 4% formaldehyde for 10 min followed by two times washing with PBS and staining with 0.1% crystal violet for 5 min. The inserts were then viewed using a Nikon TE2000 inverted microscope, using the 1× objective for observing the migratory capacity of the cells. The experiment was performed in triplicate. The cells were counted from each insert in all treatments to obtain the migration potential.

In the second experiment, the migratory capacity of cells was studied under constant exposure to drugs for 72 h. H1, H2, H3 and H10 cells were seeded at a density of 3.5 × 10^4^ cells/well in Essen BioScience ImageLock 96-well plates (cat. no. 4379, Essen BioScience Ltd., Hertfordshire, UK). After 24 h, a wound-maker tool was employed to simultaneously create a consistent wound with a uniform width across all wells. All wells were then carefully washed with preheated culture medium before drug solutions were added to the wells: 10 µM buparlisib, 10 µM trametinib or combined treatment (5 µM buparlisib and 5 µM trametinib; *n* = 6 per treatment group). Imaging was carried out every 2 h using a 10× objective in the IncuCyte^®^ Live Cell Imaging System (Essen BioScience Ltd.) and analysed to find the wound width in µm using the IncuCyte^®^ Scratch Wound Cell Migration Software Module (cat. no. 9600-0012, Essen BioScience Ltd.).

### 4.7. Apoptosis Assay

An Annexin-V flow cytometry assay was performed to assess the effects of combination treatment on apoptosis. The H1, H2, H3 and H10 lines were seeded at a density of 1 × 10^5^ cells/well in 2 mL culture medium in 6-well plates (Nunc) and allowed to reach 75% confluency. The cells were then treated with 10 µM buparlisib, 10 µM trametinib or combined therapy (5 µM buparlisib and 5 µM trametinib) for 72 h. The culture medium was then removed, the cells were washed with PBS, trypsinated using 0.25% Trypsin/EDTA, collected and centrifuged (900 rpm for 4 min). Untreated cells were maintained as controls. The supernatant was discarded, and 100 µL of Annexin V binding buffer containing 2 µL of Annexin V and propidium iodide (PI; AlexaFluor^®^488 Annexin v/dead cell apoptosis kit; Molecular Probes, Life Technologies, Waltham, MA, USA) was added to the cells and incubated for 20 min in the dark. The cells were placed on ice and further analysed using a flow cytometer (BD Fortessa, BD Bioscience, San Jose, CA, USA). Fluorescence in the FITC-A and PE-A channels were gated to a two-parameter histogram, and analysed using FloJo software (Tree Star Inc., Ashland, OR, USA). The experiment was repeated three times (*n* = 3 per experiment per treatment group). 

### 4.8. Apoptosis Array

A human apoptosis antibody array kit (R&D Systems, Inc., Minneapolis, MN, USA) was used to study the effects of treatment on 35 apoptosis-related proteins on H1 cells after 72 h. 1 × 10^6^ H1 cells were plated in 2 mL culture medium in 6-well plates (Nunc), and after reaching confluency, the cells were treated with 20 µM buparlisib, 50 nM trametinib or combined therapy (10 µM buparlisib and 25 nM trametinib) for 72 h, drug concentrations optimized for this specific experiment. Untreated wells were maintained as control. After treatment, the cells were washed and lysed using lysis buffer 17 (R&D Systems, Inc.), and a protease inhibitor was added to the lysates to prevent them from degradation. The assay was performed according to the protocol provided by the manufacturer. The arrays were developed using chemi-reagent mix (HRP substrates) provided in the kit and viewed using a ChemidocTM XRS + System (Bio-Rad laboratories AB, Oslo, Norway). The band density was analyzed using ImageJ freeware version 2.0.0-rc-68/1.52g (National Institute of Health, Bethesda, MA, USA), and graphs were plotted showing the relative intensity of band density (pixels) of the various proteins. The experiment was performed once, and each of the 35 proteins was spotted twice on the membrane.

### 4.9. Western Blot Analysis

Cell lysates from H1, H2, H3 and H10 cells treated with buparlisib and/or trametinib for 72 h were prepared for Western blot analysis. H1, H2, H3 and H10 cells were seeded as monolayers at 40–50% confluency one day prior to treatment, and then treated for 72 h with 10 μM buparlisib, 10 μM trametinib, or a combination of 5 μM buparlisib and 5 μM trametinib. Control cells were left untreated. The cells were harvested, lysed in radioimmunoassay precipitation (RIPA) buffer, and subjected to Western blotting as described previously [6]. For protein detection, the following primary antibodies were used: anti-cleaved caspase-3 (rabbit monoclonal, Cell Signaling Technology, Danvers, MA, USA, cat. #9664, 1:1000), anti-p27 Kip1 (rabbit monoclonal, Cell Signaling Technology, cat #3686, 1:1000), anti-PARP (rabbit polyclonal, Cell Signaling Technology, cat. #9542, 1:1000), anti-Bcl-2 (mouse monoclonal, NeoMarkers Fremont, CA, USA, MS-123-R1, 1:500), anti-c-IAP1 (mouse monoclonal, Santa Cruz Biotechnology, Inc., Dallas, TX, USA, sc271419, 1:100), anti-HIF-1α (mouse monoclonal, Santa Cruz Biotechnology, Inc., sc13515, 1:200), anti-PI 3-kinase p101 (rabbit polyclonal, Santa Cruz Biotechnology, Inc., sc390916, 1:200), anti-MEK1/2 (rabbit monoclonal, Cell Signaling Technology, cat. #8727, 1:1000), and anti-beta actin (rabbit polyclonal, Abcam, Cambridge, UK, ab8227, 1:2000). Quantification of protein bands was performed using ImageJ freeware version 2.0.0-rc-68/1.52g. Relative protein levels were first normalized against the loading control (beta actin) and then calculated and presented as a ratio of the untreated control. All antibodies were studied by Western blots in triplicate.

### 4.10. In Vivo Experiments

In addition, 1 × 10^6^ H1_DL2 cells in 0.1 mL PBS was injected subcutaneously in the neck region of 32 female NOD/SCID mice. After two weeks, the average tumor volume was measured to approximately 70 mm^3^. The mice were then randomized by simple randomization to daily gavage treatments of 0.1 mL vehicle (0.5% methyl cellulose with 0.2% Tween20), 50 mg/kg buparlisib, 1 mg/kg trametinib, or a combination of the two latter (25 mg/kg buparlisib + 0.5 mg/kg trametinib; *n* = 8 mice in each group). The mice were monitored daily, and caliper tumor measurements were carried out every third day. Tumor volumes were calculated using the formula [width^2^ × length]/2. The experiment was terminated after 30 days.

### 4.11. Histology and Immunohistochemistry

Paraffin-embedded tumor samples were sectioned (4 μm) and mounted on microscopic slides. Heat-induced epitope retrieval was performed in 10 mmol/L citric acid buffer at pH 7.2 preheated in a microwave and the sections were heated at 98 °C for 20 min. Sections were incubated with the primary antibody Ki67 (DAKO M7240, clone MIB-1, 1:250) at 4 °C overnight, rinsed with TBST, and incubated for 1 h at room temperature with biotin-conjugated secondary antibody, followed by 30 min incubation with ABC complex (VECTASTAIN^®^ ABC Kit, Vector Laboratories, Burlingame, CA, USA). Visualization was achieved using DAB chromogen (DAKO K3468) as the substrate, and slides were counterstained with haematoxylin (CellPath Ltd., Newtown, UK) and mounted with Entellan^®^ new (Merck Millipore, Burlington, MA, USA, 107961). Images were obtained using a Nikon TE2000 inverted microscope (Nikon Instruments Inc., Melville, NY, USA), and the percentages of Ki-67 positive cells were calculated, counting positive and negative cells using the point tool in the ImageJ freeware.

### 4.12. Statistical Analysis

The cell migration in trans-well layout was compared using Dunnett’s multiple comparisons test and the flow data was analyzed using a two-way ANOVA test with Tukey’s multiple comparisons test. The values from the apoptosis array were scaled to fold-change and compared using unpaired *t*-tests with Holm–Sidak’s correction. For the in vivo treatment experiment, the differences in endpoint tumor volumes were compared using the Mann–Whitney test. All other comparisons were carried out using unpaired, two-tailed *t*-tests. All statistical analyses were carried out using GraphPad Prism v7 (GraphPad Software, Inc.). Values presented in the figures represent means ± standard error mean (SEM). A two-tailed *p* ≤ 0.05 was considered to be statistically significant.

## 5. Conclusions

In conclusion, we show that the combined treatment with buparlisib and trametinib was superior to monotherapies in vitro, inhibiting growth and migration and inducing apoptosis in human melanoma brain metastasis cells. In our proof-of-principle in vivo study, the combinatorial approach was also more effective than monotherapies. Thus, with the improved mechanistic understanding of the treatment activity of combining buparlisib and trametinib presented here, our findings strongly encourage further evaluation of the novel combinatorial treatment approach on brain metastatic melanoma.

## Supplementary Materials

The following are available online at https://www.mdpi.com/1422-0067/20/17/4235/s1. Supplementary Figure S1: Cell survival curves with indicated IC50 concentrations of cells grown as monolayers after treatment with buparlisib, trametinib or combination. Supplementary Figure S2: Protein expression of cell lysates after in vitro treatment with 10 μM buparlisib, 10 μM trametinib or combination (5 μM + 5 μM). Supplementary Figure S3: In vitro colony formation of H2 cells pre-treated with buparlisib and trametinib. Supplementary Figure S4: Migration of brain metastatic melanoma cells during and after pre-treatment with 10 μM buparlisib, 10 μM trametinib or combination (5 μM + 5 μM). Supplementary Figure S5: Cell migration towards a chemoattractant. Supplementary Figure S6: Morphology of human melanoma brain metastatic cell lines after treatment with 10 μM buparlisib, 10 μM trametinib or combination (5 μM + 5 μM).

## Abbreviations

BBB	Blood-brain barrier
BRAF	v-Raf murine sarcoma viral oncogene homolog
EMT	Epithelial-mesenchymal transition
ERK	Extracellular signal-regulated kinase
FDA	Food and Drug Administration
MAPK	Mitogen activated protein kinase
MDPI	Multidisciplinary Digital Publishing Institute
MEK	Mitogen-activated protein kinase
NOD/SCID	Non-obese diabetic/severe compromised immunodeficiency
NRAS	Neuroblastoma RAS viral oncogene homolog
NSCLC	Non-small-cell lung cancer
PI	Propidium iodide
PI3K	Phosphoinositide 3-kinase
PTEN	Phosphatase and tensin homolog
STR	Short tandem repeat
TNFR1	Tumor necrosis factor receptor 1
