# Supplementary material for: Effective Treatment of Metastatic Melanoma by Combining MAPK and PI3K Signaling Pathway Inhibitors
Source: Int J Mol Sci. 2019 Aug 29;20(17):4235. doi: 10.3390/ijms20174235 (PMC6747502; doi:10.3390/ijms20174235)
Supplement: Supplementary file 1 [file ijms-20-04235-s001.pdf]

International Journal of *Molecular Sciences*

MDPI

Appendix A

Supplementary figures

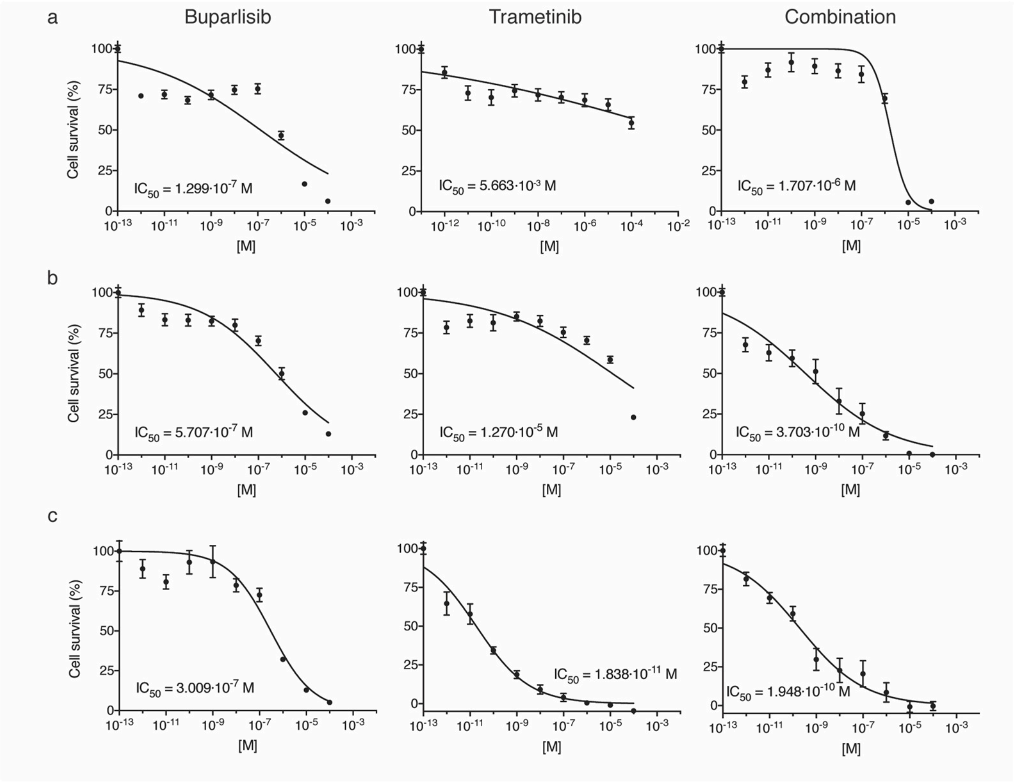

Supplementary Figure S1 Cell survival curves with indicated IC_{50} concentrations of cells grown as monolayers after treatment with buparlisib, trametinib or combination with increasing drug concentrations (0.000001 µM–100 µM). (a) $BRAF^{V600E}$ mutated H2 cells treated with buparlisib (left), trametinib (middle) and combination (right). (b) $BRAF^{L577F}$ mutated H3 cells treated with buparlisib (left), trametinib (middle) and combination (right). (c) $BRAF^{V600E}$ mutated H10 cells treated with buparlisib (left), trametinib (middle) and combination (right). The experiments were carried out in triplicate (*n* = 6 per drug per experiment).

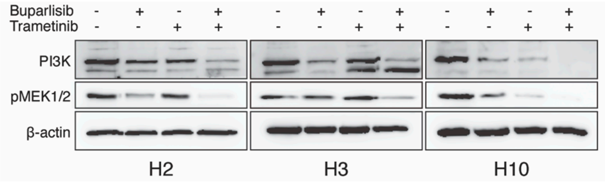

Supplementary Figure S2 Protein expression of cell lysates after *in vitro* treatment with 10 µM buparlisib, 10 µM trametinib or combination (5 µM + 5 µM). Western blots of lysates from H2 ($BRAF^{V600E}$), H3 ($BRAF^{L577F}$) and H10 ($BRAF^{V600E}$) cells after drug treatments.

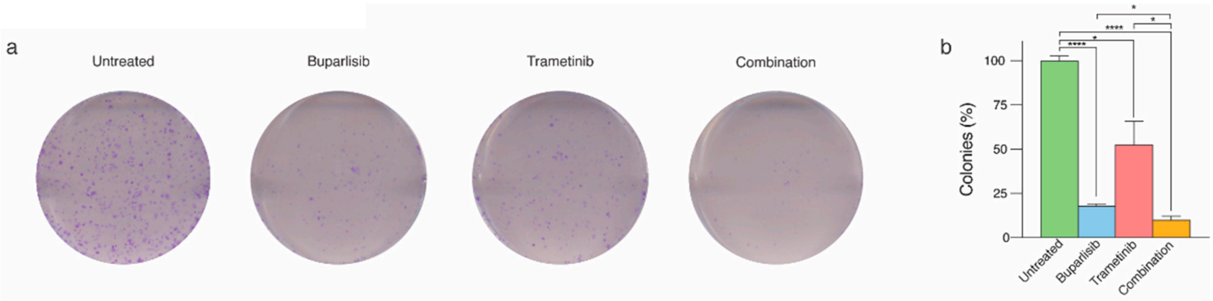

Supplementary Figure S3 In vitro colony formation of H2 cells pre-treated with buparlisib and trametinib. (**a**) Representative images of H2 cells pre-treated with 10 µM buparlisib, 10 µM trametinib or a combination (5 µM + 5 µM trametinib) grown as colonies. (**b**) The colony formation was scored and quantified as seen in the graph. The experiments were performed in triplicate. Abbreviations: *: *p* < 0.05, and ****: *p* < 0.0001.

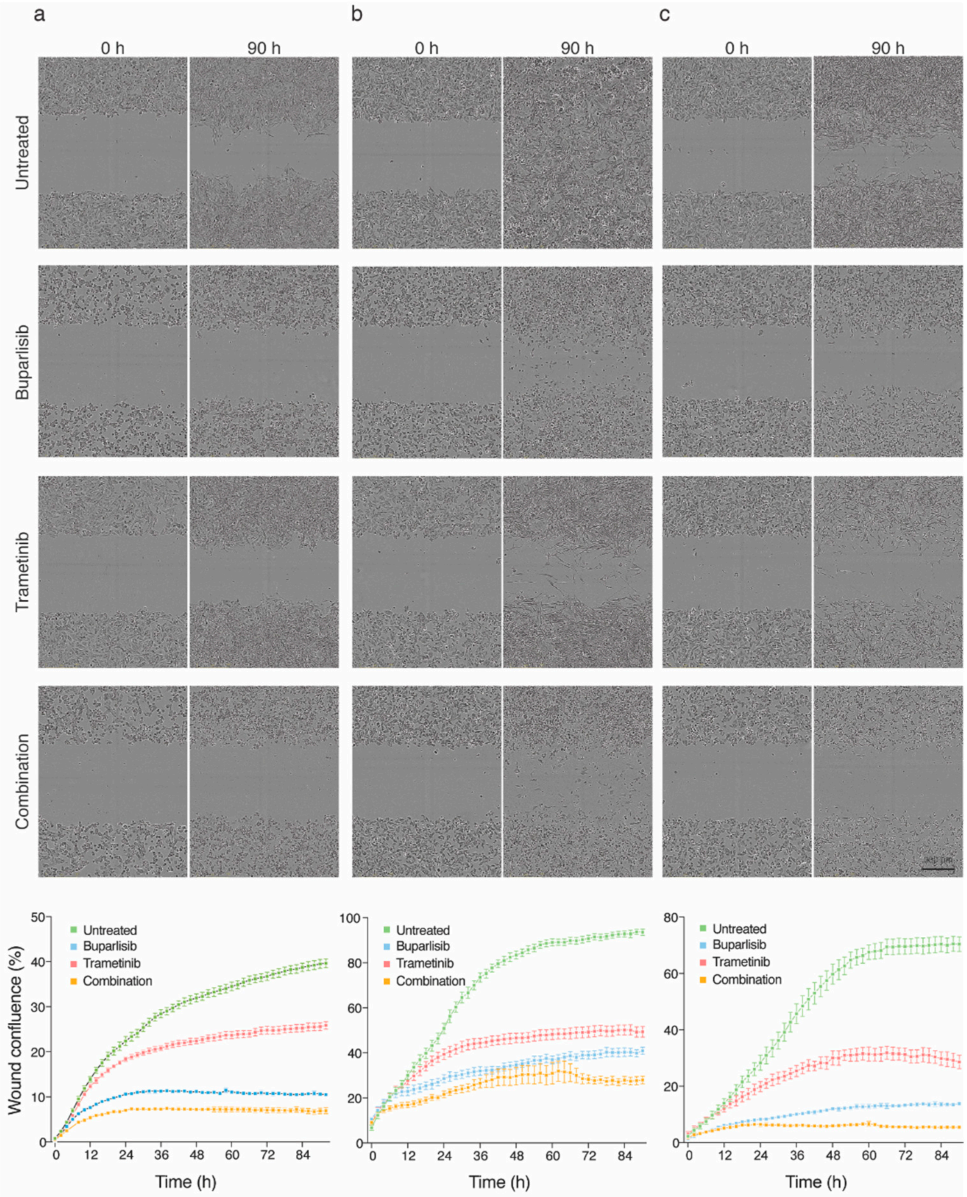

Supplementary Figure S4 Migration of brain metastatic melanoma cells during and after pre-treatment with 10 µM buparlisib, 10 µM trametinib or combination (5 µM + 5 µM). (a) Representative micrographs of H2 ($BRAF^{V600E}$), (b) H3 ($BRAF^{L577F}$) and (c) H10 ($BRAF^{V600E}$) cells before

(0 h) and at completion (90 h) of the scratch-wound experiment. The respective wound confluences were quantified and placed below each cell line (n = 6 per drug concentration). Scalebar = 300 µm.

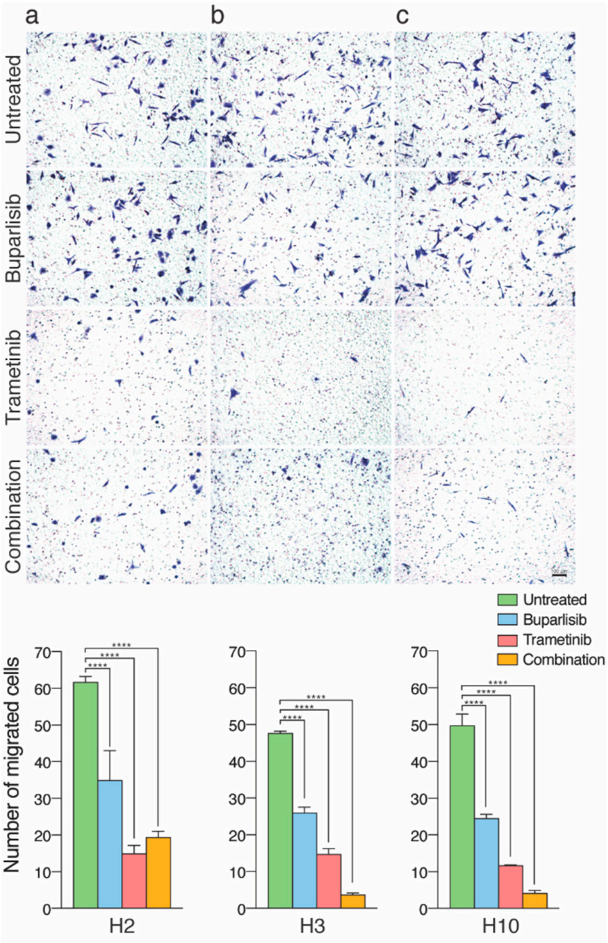

Supplementary Figure S5 Cell migration towards a chemoattractant. Representative images of migrated (**a**) H2 ($BRAF^{V600E}$), (**b**) H3 ($BRAF^{L577F}$) and (**c**) H10 ($BRAF^{V600E}$) cells after pre-treatments with 10 µM buparlisib, 10 µM trametinib or combination (5 µM + 5 µM). The number of cells were quantified based on three fields of view and placed below each cell line in the figure. The experiment was performed in triplicate. Abbreviations: ****: $p < 0.0001$.

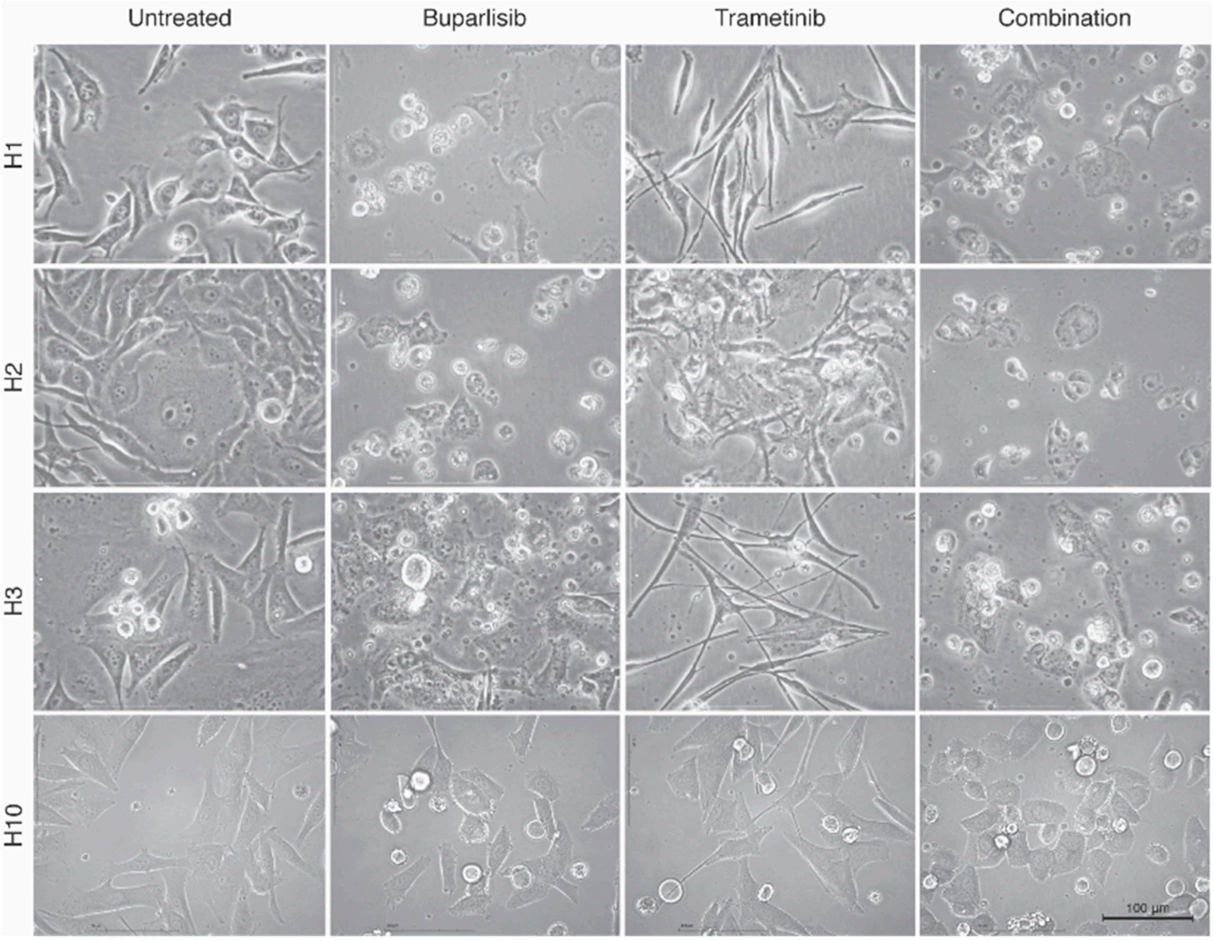

Supplementary Figure S6 Morphology of human melanoma brain metastatic cell lines after treatment with 10 μM buparlisib, 10 μM trametinib or combination (5 μM + 5 μM). Representative images of H1 ($BRAF^{V600E}$), H2 ($BRAF^{V600E}$), H3 ($BRAF^{L577F}$) and H10 ($BRAF^{V600E}$) cells can be seen from top to bottom treated with vehicle (medium), buparlisib, trametinib or a combination left to right.
